# Draft Genome Sequence of the Xanthocidin-Producing Strain *Streptomyces* sp. AcE210, Isolated from a Root Nodule of *Alnus glutinosa* (L.)

**DOI:** 10.1128/MRA.01190-18

**Published:** 2018-10-11

**Authors:** Nico Ortlieb, Nadine Keilhofer, Silvia D. Schrey, Harald Gross, Timo H. J. Niedermeyer

**Affiliations:** a Department of Microbiology and Biotechnology, Interfaculty Institute of Microbiology and Infection Medicine, University of Tübingen, Tübingen, Germany; b German Centre for Infection Research (DZIF), partner site Tübingen, Tübingen, Germany; c Department of Pharmaceutical Biology/Pharmacognosy, Institute of Pharmacy, University of Halle-Wittenberg, Halle (Saale), Germany; d Department of Physiological Ecology of Plants, Interfaculty Institute of Microbiology and Infection Medicine, Universität Tübingen, Tübingen, Germany; e IBG-2: Plant Sciences, Jülich, Germany; f Department of Pharmaceutical Biology, Pharmaceutical Institute, University of Tübingen, Tübingen, Germany; Indiana University Bloomington

## Abstract

*Streptomyces* sp. strain AcE210 exhibited antibacterial activity toward Gram-positive microorganisms and turned out to be a rare producer of the specialized metabolite xanthocidin. The 10.6-Mb draft genome sequence gives insight into the complete specialized metabolite production capacity and builds the basis to find and locate the biosynthetic gene cluster of xanthocidin.

## Abstract (precis)

*Streptomyces* sp. strain AcE210 exhibited antibacterial activity toward Gram-positive microorganisms and turned out to be a rare producer of the specialized metabolite xanthocidin.

## ANNOUNCEMENT

As part of our ongoing efforts to investigate bioactive natural products from the *Actinobacteria* (1–9), we investigated the endophytic strain *Streptomyces* sp. AcE210. It exhibited antibacterial activity toward Gram-positive bacteria, and a chemical analysis revealed its ability to produce the natural product xanthocidin (10, 11). This compound belongs to the family of cyclopentenoid antitumor antibiotics that have been clinically applied in antitumor therapy (12). However, the fact that the original producing strain, Streptomyces xanthocidicus, lost the ability to biosynthesize xanthocidin (13) precluded biotechnological production and strain optimization as well as the conduct of biosynthetic and genetic studies. With strain AcE210 in hand, biosynthetic studies can now be revisited. To locate, analyze, and prove the biosynthetic gene cluster, the sequencing of this strain was initiated.

Strain AcE210 was isolated from root nodules of *Alnus glutinosa* (L.) Gaertn. growing in Tübingen, Germany. After surface cleaning using sterile water, nodules were cracked open. Fragments were added to 50 ml of higher nitrogen content (HNC) medium (6% yeast extract, 0.05% SDS, 0.05% CaCl_2_ [pH 7.0]) and incubated at 42°C with shaking for 30 min. The suspension was filtered, and a dilution series was prepared. The filtered suspensions were plated onto International Streptomyces Project 2 (ISP-2) agar containing 5 g/liter cycloheximide, 2 g/liter nalidixic acid, and 5 g/liter nystatin. After 8 days at 27°C, strain AcE210 and eight other actinomycetes could be distinguished and separately isolated according to their morphological appearances.

DNA of strain AcE210 was harvested from cultures grown for 3 days at 27°C in 100 ml of NL410 medium (1% dextrose, 1% glycerol, 0.5% oatmeal, 1% soy flour, 0.5% yeast extract, 0.5% Bacto Casamino Acids, and 0.01% CaCO_3_ in deionized water) under agitation (180 rpm) using a Qiagen Genomic-tip 100/G kit following the manufacturer’s protocol. Subsequently, a 10-kb SMRTbell library was generated from sheared genomic DNA (gDNA) using protocols and reagents according to the manufacturers’ instructions and was subjected to sequencing using a PacBio RS II sequencing platform. Sequencing reads were processed, filtered (PreAssembler filter v1), and mapped using SMRT Analysis v2.3.0, while *de novo* assembly was performed utilizing Falcon v0.2.1 (14) with the Falcon sense options multi output, 0.70 min idt, min cov 4, local match count threshold 2, max n read 200, and n core 6 and the overlap filtering settings max diff 240, max cov 360, min cov 5, and bestn 10. The length cutoff for seed reads was set at 6,000 bp for initial mapping as well as for preassembly. Quiver v1 was used for consensus polishing using the “only unambiguously mapped reads” option. Overall, 254,702 reads (*N*_50_, 6,342 bp; mean subread length, 5,265 bp) were assembled into a 10,567,477-nucleotide draft genome at 127-fold coverage. The resulting draft genome sequence consists of 4 contigs in total with a G+C content of 70.4%. The assembled contigs were annotated with the Prokaryotic Genome Annotation Pipeline (PGAP) (15), yielding a total of 8,873 predicted protein-coding sequences. The closest related type strains based on a multilocus sequence type (MLST) analysis are Streptomyces alboflavus NRRL B-2373 and Streptomyces avermitilis DSM 46492. Automated specialized metabolism analysis using AntiSMASH v4.0.2 (16) predicted 23 biosynthetic gene clusters. The biosynthetic genes involved in xanthocidin biosynthesis will be discussed in more detail elsewhere.

### Data availability.

This whole-genome shotgun (WGS) project has been deposited in DDBJ/ENA/GenBank under the accession number QURC00000000. Raw sequencing data sets have been registered in the NCBI SRA database under the accession number SRP159767.
